# Genetic Variation in Targets of Antidiabetic Drugs and Alzheimer Disease Risk: A Mendelian Randomization Study

**DOI:** 10.1212/WNL.0000000000200771

**Published:** 2022-08-16

**Authors:** Bowen Tang, Yunzhang Wang, Xia Jiang, Madhav Thambisetty, Luigi Ferrucci, Kristina Johnell, Sara Hägg

**Affiliations:** From the Department of Medical Epidemiology and Biostatistics, Karolinska Institutet, Stockholm (B.T., Y.W., K.J., S.H.); Department of Clinical Neuroscience, Karolinska Institutet, Stockholm (X.J.); Brain Aging and Behavior Section, National Institute on Aging (M.T.); and Longitudinal Studies Section (L.F.), National Institute on Aging.

## Abstract

**Background and Objectives:**

Previous studies have highlighted antidiabetic drugs as repurposing candidates for Alzheimer disease (AD), but the disease-modifying effects are still unclear.

**Methods:**

A 2-sample mendelian randomization study design was applied to examine the association between genetic variation in the targets of 4 antidiabetic drug classes and AD risk. Genetic summary statistics for blood glucose were analyzed using UK Biobank data of 326,885 participants, whereas summary statistics for AD were retrieved from previous genome-wide association studies comprising 24,087 clinically diagnosed AD cases and 55,058 controls. Positive control analysis on type 2 diabetes mellitus (T2DM), insulin secretion, insulin resistance, and obesity-related traits was conducted to validate the selection of instrumental variables.

**Results:**

In the positive control analysis, genetic variation in sulfonylurea targets was associated with higher insulin secretion, a lower risk of T2DM, and an increment in body mass index, waist circumference, and hip circumference, consistent with drug mechanistic actions and previous trial evidence. In the primary analysis, genetic variation in sulfonylurea targets was associated with a lower risk of AD (odds ratio [OR] = 0.38 per 1 mmol/L decrement in blood glucose, 95% CI 0.19–0.72, *p* = 0.0034). These results for sulfonylureas were largely unchanged in the sensitivity analysis using a genetic variant, *rs757110*, that has been validated to modulate the target proteins of sulfonylureas (OR = 0.35 per 1 mmol/L decrement in blood glucose, 95% CI 0.15–0.82, *p* = 0.016). An association between genetic variations in the glucagon-like peptide 1 (GLP-1) analogue target and a lower risk of AD was also observed (OR = 0.32 per 1 mmol/L decrement in blood glucose, 95% CI 0.13–0.79, *p* = 0.014). However, this result should be interpreted with caution because the positive control analyses for GLP-1 analogues did not comply with a weight-loss effect as shown in previous clinical trials. Results regarding other drug classes were inconclusive.

**Discussion:**

Genetic variation in sulfonylurea targets was associated with a lower risk of AD, and future studies are warranted to clarify the underlying mechanistic pathways between sulfonylureas and AD.

Alzheimer disease (AD) is a neurodegenerative disease, characterized by aberrant protein aggregation and neuronal loss in the brain that leads to cognitive decline, memory loss, and ultimately death.^1^ However, currently, only a few agents or drugs that can improve symptoms have been approved for AD, whereas their neuroprotective effects remain uncertain.^2^ Developing new drugs for AD is imperative but also extremely challenging with more than 400 candidates recently failed in phase III trials.^3^ Drug repurposing or repositioning, where approved drugs are tested for a novel indication, has been proposed as a more rapid and cost-effective strategy to identify potential AD treatments because approved drugs possess well-documented information for mechanism of actions and comprehensive safety profiles.^4^

AD and type 2 diabetes mellitus (T2DM) are 2 of the most prevalent diseases in the aged population. A meta-analysis of 1,746,777 participants reported a 53% higher risk of developing AD in patients with T2DM.^5^ Besides, AD has been proposed as “type 3 diabetes” with insulin resistance and impaired glucose control in the brain.^6^ Antidiabetic drugs, based on their original intention of enhancing insulin signaling and regulating glucose metabolism, have been highlighted as repurposing candidates for AD.^7^ Several randomized clinical trials (RCTs) have been conducted in patients with early or mild-to-moderate AD to investigate the disease-modifying effects of antidiabetic drugs, but the evidence to date was inconclusive.^8^ Given the long prodromal phase of AD, clinical trials targeting early or mild-to-moderate AD have been considered belated, whereas primary intervention in preclinical AD or even earlier may offer the best opportunity of therapeutic success.^9^ However, such primary prevention trials are challenging because they require considerably large sample sizes and long-duration intervention.

Mendelian randomization (MR) is a statistical tool using genetic variants as instrumental variables (IVs) to make causal inference between exposure(s) and outcome(s). Because genetic variants are assigned randomly at conception and before disease onset, MR is considered as a “natural” RCT, which can minimize confounding and reverse causation.^10^ Particularly for the genetic variants within the genes that encode drug target proteins, such variants may influence the expression of genes, modulate the function of encoded proteins, and thereby closely proxy drug mechanism actions. In the spirit of natural RCTs, MR studies leveraging such druggable variants are useful in identifying drug repurposing opportunities and predicting side effects.^11^ An MR study used a variant on the *HMGCR* gene to proxy statin use and found that genetically mimicked statin use was associated with a higher risk of T2DM, consistent with the evidence from an RCT of 129,170 participants.^12^ Apart from controlling for confounding and reverse causality, MR also provides the possibility to emulate primary prevention trials that comprise large sample sizes (recent genetic discoveries are usually based on hundreds of thousands of participants) and long intervention duration (genetically instrumented exposure occurs before the outcome and is lifelong). Hence, we conducted an MR study to examine the effects of genetic variation in antidiabetic drug targets on AD risk.

## Methods

### Study Design

The current study was conducted using a 2-sample MR design, which extracted exposure and outcome data from 2 independent nonoverlapping populations. Genetic variants within the genes that encode protein targets of antidiabetic drugs (*cis*-variants) were identified in a genome-wide association study (GWAS) summary dataset for blood glucose and used as proxy for antidiabetic drug use. Lowering blood glucose is an established physiologic response to antidiabetic drug treatment, and hence, blood glucose is the biomarker of interest in our study. To retain the validity of causal estimation, 3 MR model assumptions are essential, which are (1) a robust association between IVs and target proteins (relevance), (2) independence of IVs on confounders (exchangeability), and (3) no direct effects of IVs on AD risk other than through the drug targets (exclusion restriction). A framework of our study design is presented in eFigure 1, links.lww.com/WNL/C91.

### Blood Glucose GWAS Data

IV-exposure associations were extracted from a GWAS of blood glucose analyzed on participants of European ancestry from UK Biobank (UKB).^13^ Individuals with a diagnosis of diabetes in the inpatient registry (defined as E10-14 in *ICD-10* and 2500-2529 in *ICD-9*) or with self-reported diabetes in questionnaires were excluded from the analysis. In the association testing, a mixed linear model–based method was used to control for population stratification by principle components and relatedness by a genetic relationship matrix.^14^ Finally, 326,885 participants were analyzed. Details about the GWAS are provided in eAppendix 1, links.lww.com/WNL/C91.

### AD GWAS Data

The AD summary statistics (IV-outcome associations) were extracted from a previously conducted GWAS.^15^ In phase 1, the data from Alzheimer's disease working group of the Psychiatric Genomics Consortium, the International Genomics of Alzheimer's Project, and the Alzheimer's Disease Sequencing Project were meta-analyzed, totaling 24,087 clinically diagnosed late-onset AD cases and 55,058 controls. In phase 3, 47,793 AD-by-proxy cases and 328,320 controls from UKB were additionally meta-analyzed on top of the phase 1 stage, resulting in 71,880 AD/AD-by-proxy cases and 383,378 controls. We used the dataset that only contains clinically diagnosed AD cases in primary analysis and the dataset that contains AD/AD-by-proxy cases in sensitivity analysis.

### Instrument Selection

Seven major classes of antidiabetic drugs were initially identified, including metformin, dipeptidyl peptidase 4 (DPP-4) inhibitor, sodium-glucose cotransporter 2 (SGLT2) inhibitor, insulin/insulin analogues, glucagon-like peptide 1 (GLP-1) analogues, sulfonylureas, and thiazolidinediones (TZD).^16^ Information regarding the pharmacologically active protein targets and corresponding encoding genes was retrieved from the DrugBank and the ChEMBL databases separately (Table 1).^17,18^ Because the protein targets of metformin differed in the 2 databases and the molecules underlying metformin's physiologic effects remain largely unknown,^19^ metformin was excluded from further analysis.

**Table 1:**
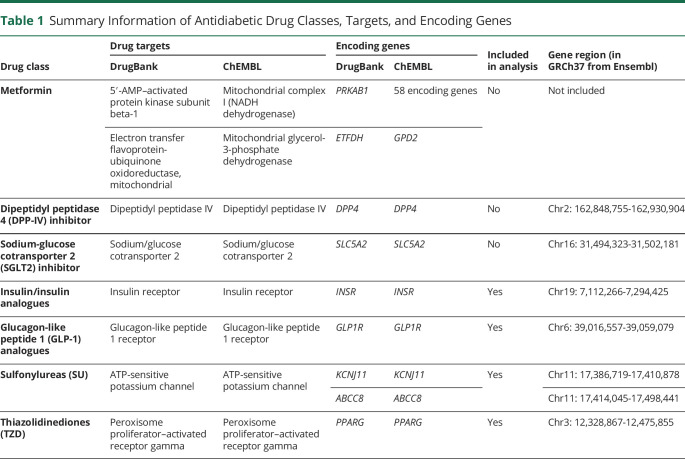
Summary Information of Antidiabetic Drug Classes, Targets, and Encoding Genes

**Figure 1:**
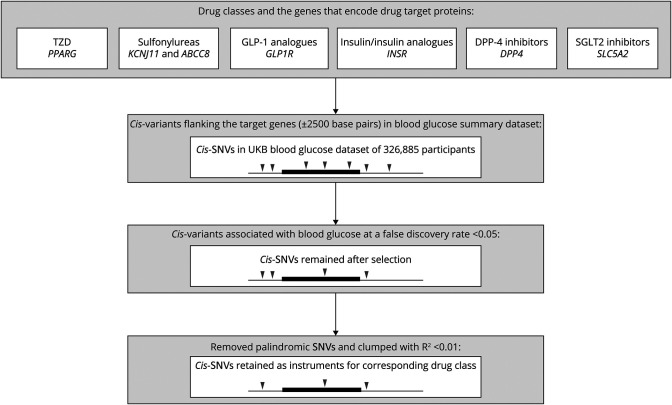
Instrument Selection for Antidiabetic Drug Classes Black line represents DNA strand, and raised box represents the target gene region. Wedges represent specific genetic variants (SNVs). Disappearing of wedges indicates the exclusion of SNVs. DPP-4 inhibitors = dipeptidyl peptidase 4 inhibitors; GLP-1 analogues = glucagon-like peptide 1 analogues; SGLT2 inhibitors = sodium-glucose cotransporter 2 inhibitors; SNVs = single-nucleotide variations; TZD = thiazolidinediones; UKB = UK Biobank.

Next, we identified the *cis*-variants within each encoding gene (±2,500 base pairs of the gene location) and retained the variants associated with blood glucose at a false discovery rate of <0.05. None of the variants for SGLT2 inhibitors survived the selection, hence being excluded from further analysis. Palindromic single-nucleotide variations (SNVs [formerly SNPs]; SNVs with the same pair of purine pyrimidine bases on forward and reverse strands) were excluded to avoid ambiguity in the identification of effect alleles. The 2 variants for DPP4 inhibitors are both palindromic (no high-LD proxies were found), so the drug was excluded from further analysis. The variants that remained for each drug class were then clumped with a R^2^ of 0.01 and a window size of 500 kB, complemented with a sensitivity analysis clumping with R^2^ from 0.01 to 0.50 to gain greater precision by including a larger number of partially independent variants.^11^ The process of instrument selection is displayed in Figure 1. In addition, we consulted the literature and identified 1 additional variant, *rs757110*, for sulfonylureas, which has been validated as a strong proxy in in vitro and population studies.^20,21^

### Positive Control Analysis

To validate our selection of IVs, positive control analysis was performed with T2DM, insulin secretion, insulin resistance, and obesity-related traits as outcomes. T2DM is the original indication of antidiabetic drugs, whereas sulfonylureas and GLP-1 analogues promote insulin secretion and TZD increases insulin sensitivity.^16^ IVs for insulin/insulin analogues were selected within *INSR* that encodes the insulin receptor, so it would be expected to alter the function of the insulin receptor and reduce insulin resistance. Obesity is another phenotype influenced by antidiabetic drugs. A meta-analysis of clinical trials suggests that insulin analogues, sulfonylureas, and TZD contribute to weight gain, and GLP-1 analogues cause weight loss.^22^ Hence, 3 obesity-related traits, including body mass index (BMI), waist circumference (WC), and hip circumference (HIP), were used as outcomes.

Because the GWAS datasets for insulin secretion, insulin resistance, WC, and HIP (outcome datasets) contain less SNVs than the UKB blood glucose GWAS (exposure dataset), we retained nonpalindromic SNVs available in both datasets and performed clumping to obtain IVs for the positive control analyses (R^2^ < 0.01). Genetically predicted drug effects that showed directional consistency with clinical trial evidence/drug mechanisms were considered to pass the positive control analysis.

In addition to the positive control analyses, we also explored the association between genetic variation in antidiabetic drug targets and cardiovascular diseases/hippocampal volume despite rather inconclusive evidence given by previous clinical trials.^23-28^ The details about these analyses and results are provided in eAppendix 2, links.lww.com/WNL/C91. Information for the GWAS datasets used in our study is summarized in eTable 1, links.lww.com/WNL/C91.^29-33^ Characteristics of the SNVs retained as IVs for each analysis are described in eTables 2 and 3, links.lww.com/WNL/C91.

### Standard Protocol Approvals, Registrations, and Patient Consents

Our analysis of UKB data has been conducted under application number “22224.” The summary statistics for AD, BMI, and T2DM do not contain any personal information, and the GWAS have obtained ethical approval from relevant ethics review boards.

### Statistical Analysis

First, the IV-exposure association from the blood glucose GWAS dataset and the IV-outcome association from the outcome GWAS dataset were merged. The causal association was estimated with the Wald ratio test for 1 single IV and with the random-effects inverse variance–weighted (IVW) method for multiple IVs.^10^ The IVW method provides unbiased causal estimation when all IVs are valid or if overall pleiotropy is balanced to be zero.^10^ The Cochran's Q test was performed to test heterogeneity within IVs and to detect any types of pleiotropy, balanced or unbalanced (*p* for heterogeneity <0.05).^10^ To deal with unbalanced pleiotropy, the MR-Egger regression was applied. MR-Egger provides reliable causal estimation even if all IVs are invalid and indicates the presence of unbalanced pleiotropy at *p* for intercept <0.05.^10^ Strength of the IVs was tested by F statistics and indicates weak instruments when F statistics <11. All analyses were conducted using the “TwoSampleMR” package in R software version 3.6.0.^34^ To resemble the lowering effects of blood glucose by antidiabetic drugs, all estimations were scaled to per 1 mmol/L decrement in blood glucose. A Bonferroni-corrected significance level of *p* value < 0.012 (0.05/4) was used to adjust for multiple testing of 5 drug classes. For the genetically instrumented drug class confirmed to be associated with AD, we further investigated the colocalization between blood glucose and AD within the target gene region using the “coloc” package.^35^

### Data Availability

Genetic datasets for AD/AD-by-proxy, T2DM, insulin secretion, insulin resistance, BMI, WC, and HIP are publicly available through the link listed in eTable1, links.lww.com/WNL/C91. The UKB data are available through application. Scripts are available on personal request (email: bowen.tang@ki.se).

## Results

### Positive Control Analyses

**Figure 2:**
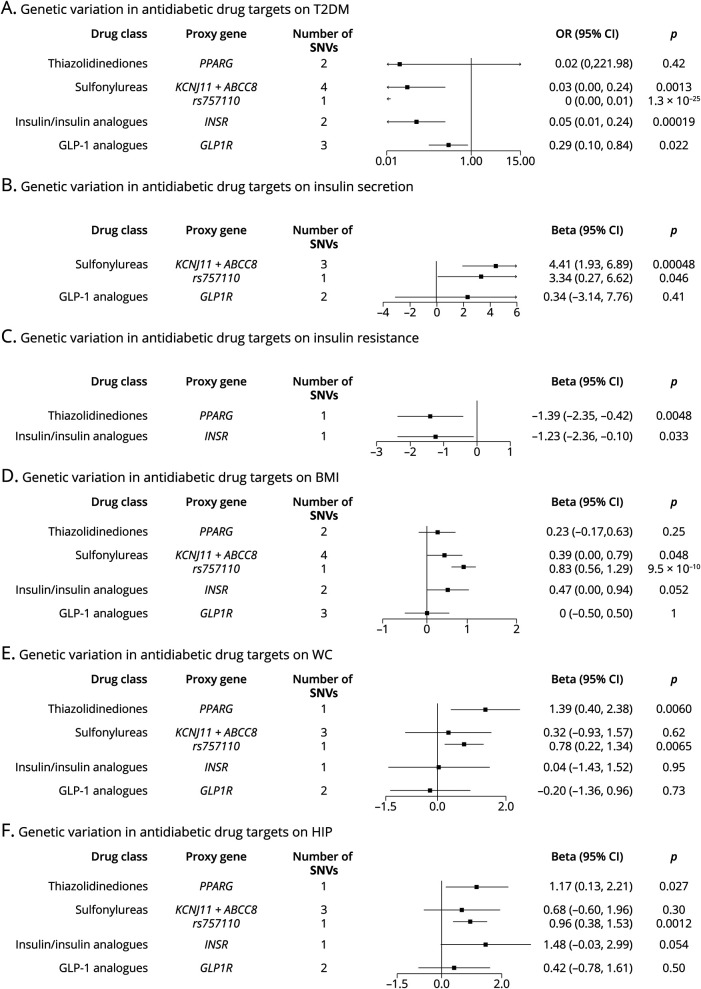
Estimated Effects of Genetic Variation in Antidiabetic Drug Targets on Glucose Metabolism–Related Traits (Panel A–C) and Obesity-Related Traits (Panel D–F) Proxy gene is the gene that encodes the drug target protein, and *rs757110* is the variant that has been validated to modulate the protein target of sulfonylureas. All the estimates were scaled to per 1 mmol/L decrement in blood glucose. The estimated beta coefficients on BMI, WC, and HIP are expected to be in agreement with the evidence from clinical trials, that is, taking insulin analogues, sulfonylureas, and TZD contributes to weight gain, and taking GLP-1 analogues causes weight loss. BMI = body mass index; GLP-1 analogues = glucagon-like peptide 1 analogues; HIP = hip circumference; IVs = instrumental variables; OR = odds ratio; SNVs = single-nucleotide variations; T2DM = type 2 diabetes mellitus; TZD = thiazolidinediones; WC = waist circumference.

As shown in Figure 2, panel A, genetic variation in the targets of TZD, sulfonylureas, insulin analogues, and GLP-1 analogues was associated with a decreased risk of T2DM. When looking into insulin secretion and insulin resistance (Figure 2, panels B and C), genetic variation in the targets of sulfonylureas and GLP-1 analogues was associated with increased insulin secretion, whereas insulin/insulin analogues and TZD were associated with decreased insulin resistance, consistent with the drug mechanism of actions. For obesity-related traits (Figure 2, panels D–F), the estimates for TZD and sulfonylureas suggested increment in BMI, WC, and HIP, consistent with evidence from the meta-analysis of clinical trials. However, the estimates for insulin/insulin analogues and GLP-1 analogues were varying across the 3 obesity-related traits.

### Effects of Genetic Variation in Antidiabetic Drug Targets on AD Risk

**Figure 3:**
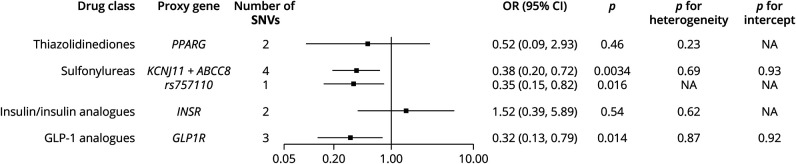
Estimated Effects of Genetic Variation in Antidiabetic Drug Targets on Alzheimer Disease and Results From the Primary Analysis Using a GWAS Dataset Comprising 24,087 Clinically Diagnosed Late-Onset AD Cases Proxy gene is the gene that encodes the drug target proteins, and *rs757110* is the variant that have been validated to modulate the protein targets of sulfonylureas. *p* < 0.01 indicates statistical significance, and 0.01 < *p* < 0.05 indicates suggestive significance. *p* for heterogeneity <0.05 indicates possible pleiotropy, whereas *p* for intercept <0.05 indicates substantial bias from pleiotropy. All the ORs were scaled to per 1 mmol/L decrement in blood glucose. NA indicates that the Cochran's Q test (heterogeneity test) or the MR-Egger regression (intercept test) is not available because of the limited number of IVs. AD = Alzheimer disease; GLP-1 analogues = glucagon-like peptide 1 analogues; GWAS = genome-wide association study; IVs = instrumental variables; MR = mendelian randomization; OR = odds ratio; SNVs = single-nucleotide variations; TZD = thiazolidinediones.

The results from our primary analysis using a GWAS dataset that contains 24,087 clinically diagnosed late-onset AD cases are shown in Figure 3. Generally, genetic variation in sulfonylurea targets was associated with a reduced risk of AD at the Bonferroni-corrected threshold (odds ratio [OR] = 0.38 per 1 mmol/L decrement in blood glucose, 95% CI 0.19–0.72, *p* = 0.0034). The association was consistent in the analysis using the validated proxy, *rs757110* (OR = 0.35, 95% CI 0.15–0.82, *p* = 0.016). Meanwhile, the estimates for sulfonylureas in the weighted median method (OR = 0.38, 95% CI 0.17–0.84) and MR-Egger regression (OR = 0.42, 95% CI 0.05–3.79) also confirmed a reduced risk of AD, although with a wider CI as the MR-Egger regression produces larger standard errors than the conventional IVW method (eTable 4, links.lww.com/WNL/C91). Besides, a suggestive association between genetic variation in the GLP-1 analogue target and lower risk of AD was observed across different MR methods (Figure 3 and eTable 4, links.lww.com/WNL/C91, IVW: OR = 0.32, 95% CI 0.13–0.79, *p* = 0.014; weighted median: OR = 0.34, 95% CI 0.11–1.07, *p* = 0.066; MR-Egger: OR = 0.35, 95% CI 0.05–2.52, *p* = 0.49). For all the estimates, no heterogeneity within IVs or substantial pleiotropy was detected (Figure 3, *p* for heterogeneity >0.05, *p* for pleiotropy >0.05).

**Figure 4:**
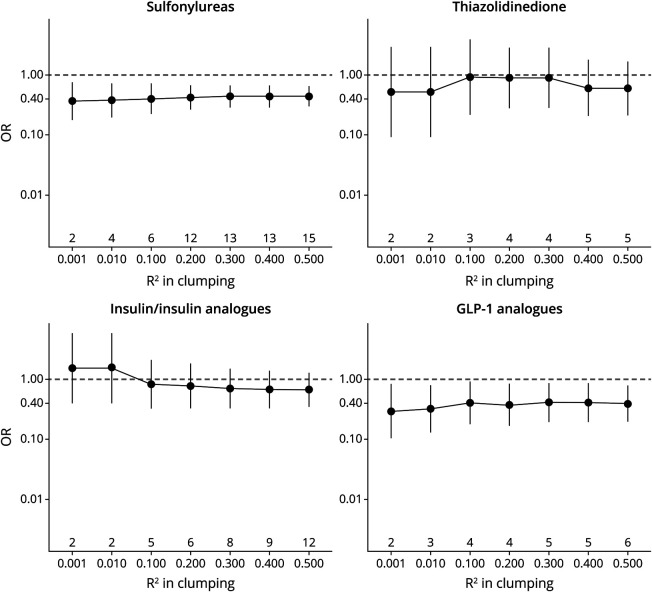
Estimated Effects of Genetic Variation in Antidiabetic Drug Targets on Alzheimer Disease and Results From the Sensitivity Analysis Using a GWAS Dataset Comprising 24,087 Clinically Diagnosed Late-Onset AD Cases and Clumping the cis-Variants With R2 From 0.001 to 0.50 The numbers below the x axis are the value of R^2^, and the numbers above the x axis are the number of IVs after clumping with the corresponding R^2^. AD = Alzheimer disease; GLP-1 analogues = glucagon-like peptide 1 analogues; GWAS = genome-wide association study; IVs = instrumental variables; OR = odds ratio.

In the sensitivity analysis using a GWAS dataset comprising 71,880 AD/AD-by-proxy cases (eFigure 2, links.lww.com/WNL/C91), genetic variation in the targets of sulfonylureas and GLP-1 analogues showed protective effects (sulfonylureas: OR = 0.78, 95% CI 0.58–1.05; GLP-1 analogues: OR = 0.53, 95% CI 0.37–0.77), although the effect sizes attenuated toward the null possibly because of the dilution effects by AD-by-proxy cases. Considering the possible uncertainty arising from instrumenting each drug class with a relatively small number of IVs, we conducted a sensitivity analysis retaining more partially independent IVs by clumping the variants with relaxing R^2^. The estimates for sulfonylureas and GLP-1 analogues stabilized throughout the R^2^ ranging from 0.001 to 0.50; meanwhile, the standard error decreased with more IVs being included. However, the estimates insulin analogue and TZD varied across different R^2^ and included the null (Figure 4).

### Colocalization Analysis for Sulfonylureas and GLP-1 Analogues

Colocalization analysis was performed for sulfonylureas and GLP-1 analogues within the drug target encoding genes (±2,500 base pairs of *KCNJ11* and *ABCC8* for sulfonylureas and of *GLP1R* for GLP-1 analogues). The results are shown in eTable 5, links.lww.com/WNL/C91. Generally, we did not observe strong evidence suggesting colocalization between blood glucose and AD within the 2 gene regions for sulfonylureas (probability for sharing 1 common causal variant: 2.5% in *KCNJ11* and 3.9% in *ABCC8*); however, when looking into the regional association plots (eFigure 3, links.lww.com/WNL/C91), a trend of colocalization was noted. For GLP-1 analogues, we did not find any evidence of either sharing a common variant or a distinct trend of colocalization within *GLP1R* (eTable 5 and eFigure 4, links.lww.com/WNL/C91). For more details regarding the colocalization analyses and results, please see eAppendix 3, links.lww.com/WNL/C91.

## Discussion

We investigated the effects of genetic variation in antidiabetic drug targets on AD risk using a combination of genetic datasets for blood glucose (∼300,000 participants) and AD (24,087 AD cases and 55,058 controls). We found evidence to support that genetic variation in sulfonylurea targets was associated with a lower risk of AD.

A handful of observational studies have investigated the relationship of sulfonylureas with AD, but the results are rather inconclusive.^36-39^ In a prospective study following 127,209 dementia-free individuals for 7 years, a marginal protective effect was observed in diabetic patients (sulfonylureas vs not taking any antidiabetic drugs: HR = 0.85, 95% CI 0.71–1.01).^38^ Conversely, in a case-control study of 7,086 AD cases, sulfonylureas were not associated with the development of AD (users vs nonusers: OR = 1.01, 95% CI 0.72–1.42).^37^ The present MR study differs from these observational studies in 3 key aspects. First, the observational studies were conducted in diabetic patients and hence were unable to separate the true drug effect from that of diabetes, i.e., confounding by indication. Meanwhile, the present MR study investigated the genetically predicted drug effects in a nondiabetic general population. Second, the observational studies measured drug use at baseline, but the existence of poor monotherapy adherence may cause contamination. A study on veterans showed that around 40% of diabetic patients who initiated sulfonylureas added or switched to metformin afterward.^39^ Drug adherence is less of a concern in our MR models because the genetically instrumented exposure is lifelong. Finally, unmeasured confounding could also be an issue in observational studies. Although the observational studies have controlled for confounders available in their datasets, these are difficult to measure, or even unknown, and may linger and induce bias. Conversely, taking advantage of the random allocation of genetic variants at conception, MR is expected to be less affected by confounding.^10^

Sulfonylureas lower blood glucose by blocking the K_ATP_ channel on the membrane of pancreatic beta cells. Physiologically, the pancreatic K_ATP_ channel closes as a response to blood glucose increase, subsequently producing a membrane depolarization and stimulating insulin secretion.^20^ Therefore, sulfonylurea use might relate to AD partly through its regulatory effects on glucose metabolism. Evidence from a previous MR study showed that increased fasting blood glucose (OR = 1.33, 95% CI 1.04–1.68) and β-cell dysfunction (OR = 1.92, 95% CI 1.15–3.21) contributed to a higher risk of AD.^40^ Particularly, AD has been proposed as a brain-specific form of diabetes, a “type 3 diabetes.”^6^ A study by An et al.^41^ found that abnormalities in brain glucose homeostasis, characterized by higher brain blood glucose levels, reduced glycolytic flux, and lower neuronal glucose transporter 3 levels, were intrinsic to AD pathogenesis. Moreover, periphery glucose regulation might affect the glucose homeostasis in the CNS. The same study showed that longitudinal increases in fasting plasma glucose levels were associated with higher brain tissue glucose concentrations.^41^

The K_ATP_ channel is also abundantly expressed in the brain.^42^ Although the exact role of K_ATP_ channels in the brain is largely unknown, it has been found to modulate neurotransmitter release and to mediate the action of memantine in the mouse hippocampus.^42-44^ Besides, the brain K_ATP_ channel has been evident to affect CNS function by clinical investigations. A study compared neuropsychological features between adults with permanent neonatal diabetes mellitus (PNDM) caused by *KCNJ11* variations and by *INS* variations (the gene encodes insulin; its variations influence insulin synthesis). Patients with *KCNJ11* variations were more likely to have abnormal CNS features, such as learning difficulties, reduced IQ, and motor deficits, whereas patients with *INS* variations rarely showed neurologic abnormalities.^45^ Furthermore, Clark et al.^46^ have confirmed that such neurologic abnormities originate from the CNS. Analogous to the genetic variations in patients with PNDM, our IVs selected within the *KCNJ11* and *ABCC8* genes may simultaneously capture the modulation of the K_ATP_ channel in the brain and pancreas. This synthesis would incorporate the downstream effects, either through neurologic alterations or blood glucose regulation, in our MR models. Given the poor penetration of the sulfonylurea across the blood-brain barrier,^47^ caution is warranted in repurposing the existing sulfonylurea agents for AD, especially until the elucidation of the underlying mechanistic pathways, pharmacokinetics, and pharmacodynamics.

A suggestive association between genetic variation in GLP-1 analogue targets and lower risk of AD was also observed in our study. GLP-1 lowers blood glucose by binding to GLP-1 receptors on pancreatic β-cells and stimulating insulin production and secretion.^48^ GLP-1 crosses the blood-brain barrier, and GLP-1 receptors are also found in the CNS.^48^ In vivo studies have found that GLP-1 analogues increased hippocampal neuronal density, protected against synaptic dysfunction, and reduced hyperphosphorylated tau.^49^ In an RCT following 38 patients with AD for 26 weeks, liraglutide, a GLP-1 analogue, has been found to prevent the decline of brain glucose metabolism compared with placebo but produce no effect on cognition or Aβ load, although the study was underpowered to detect such changes.^50^ Besides, 2 ongoing RCTs (phase 2 and phase 3) are planning to recruit patients with early AD to examine the effects of GLP-1 analogues (liraglutide and semaglutide, respectively) on cerebral glucose metabolism, cognitive performance, and pathologic biomarkers (ClinicalTrials.gov Identifier: NCT01843075 and NCT04777396), which is expected to provide more convincing evidence to this topic.

The present MR study has several strengths. First, the participants were restricted to people of European ancestry, which minimized the possible bias arising from population stratification. Second, the IVs were selected from the *cis*-variants within the coding genes for drug targets (±2.5 kB), which controlled the likelihood of pleiotropy by IVs tagging other genes. Also, no heterogeneity within the IVs was detected in our MR analysis, further substantiating the absence of pleiotropy that would bias our estimations. Third, we conducted a set of positive control analyses to validate the strength of our IVs. The results for sulfonylureas, instrumented either by the 4 clumped IVs or by the validated proxy *rs757110*, were in line with the evidence from prior meta-analyses of clinical trials. This strongly supported these IVs as appropriate proxies. Then, a reduced risk in AD was observed, consistent in these 2 sets of IVs, which further lends support for a putative causal relationship between sulfonylureas and AD.

The study has several limitations. First, our study can only predict the on-target effects of antidiabetic drugs because only the well-documented protein targets were included in our analysis. Drug effects that are not exerted through these protein targets (off-target effects) cannot be captured in our MR models. Second, the genetically predicted drug effects may be somewhat different from therapeutic practice. An exposure instrumented by genetic variants is present from birth and lasts for a lifetime. Our analyses may therefore be interpreted as assessing long-term modulation effects of drug target proteins. Moreover, given that genetic effects are lifelong, our estimates cannot reflect the effects of exposure to antidiabetic drugs during a certain period of life. Third, metformin, SGLT2 inhibitors, and DPP4 inhibitors were excluded from the analysis because of yet unclear mechanism of actions or lack of proper IVs. Also, our results regarding insulin/insulin analogues, TZD, and GLP-1 analogues are inconclusive, likely because of the uncertainty by the small number of IVs. Future studies are warranted to reexamine these drug classes when more or stronger IVs are available. Besides, because we only used the genetic summary data restricted to the population of European ancestry, the generalizability of our results would be confined in European ancestral populations. Finally, we did not find strong colocalization evidence for blood glucose and AD within *KCNJ11* and *ABCC8* for sulfonylureas, which might be due to weak variant-AD association in the region. Therefore, our findings regarding sulfonylureas warranted further examinations when a larger AD GWAS is available.

In conclusion, this study provides supportive evidence for genetic variation of sulfonylurea targets, through the modulation of the K_ATP_ channel, which was associated with a lower risk of AD. Future studies should be conducted to clarify the underlying mechanistic pathways between sulfonylureas and AD. The study also exemplifies how the MR design may be a promising tool for finding new indications for approved drugs. The method allows testing interventions that are otherwise costly, time-consuming, or, in other ways, impractical and should be considered a screening instrument in the drug development phase.

## Glossary

AD: Alzheimer disease
BMI: body mass index
DPP-4: dipeptidyl peptidase 4
GLP-1: glucagon-like peptide 1
GWAS: genome-wide association study
HIP: hip circumference
IVs: instrumental variables
IVW: inverse variance–weighted method
MR: mendelian randomization
PNDM: permanent neonatal diabetes mellitus
RCTs: randomized clinical trials
SGLT2: sodium-glucose cotransporter 2
SNV: single-nucleotide variation
T2DM: type 2 diabetes mellitus
TZD: thiazolidinediones
UKB: UK Biobank
WC: waist circumference
